# The impact of zwitterionic surfactants on optode-based nanosensors *via* different fabrication approaches and sensing mechanisms†

**DOI:** 10.1039/d4an00687a

**Published:** 2024-07-26

**Authors:** Adrian A. Mendonsa, Tyler Z. Sodia, Kevin J. Cash

**Affiliations:** a Chemical and Biological Engineering Department, Colorado School of Mines Golden CO 80401 USA kcash@mines.edu; b Quantitative Biosciences and Engineering Department, Colorado School of Mines Golden CO 80401 USA

## Abstract

In this work, we explored the impact of zwitterionic surfactants, sulfobetaine 16 (SB-16) and a PEG-phospholipid conjugate (DSPE-PEG), on nanosensor performance. We fabricated four sensors (for Na^+^, K^+^, Al^3+^, and O_2_) and examined how these surfactants influenced various aspects, including fabrication methods, sensing mechanisms, and the incorporation of nanomaterials. Our results highlighted SB-16's role in enhancing selectivity in ion-exchange sensors (Na^+^ and K^+^) while maintaining sensitivity akin to its PEG counterpart. The liquid–liquid extraction based sensors (Al^3+^) were unaffected by surfactant choice, while the O_2_ sensors that operate *via* collisional quenching exhibited reduced sensitivity with SB-16 when compared to its PEG-based counterpart. Additionally, the SB-16 sensors proved adaptable to different fabrication approaches (SESE – single emulsion solvent evaporation and FNP – flash nanoprecipitation), showcased good cell viability and maintained a functional lifetime of at least five days. Furthermore, *via* the use of quantum dots, we showed that it is possible to incorporate other nanomaterials into the sensing phase of SB-16 sensors. Future investigations could target enhancing the pH stability of zwitterionic surfactants to further advance their applicability in sensor technologies.

## Abstract 2

We studied the impact of zwitterionic surfactants on three different sensing mechanisms, nanomaterial encapsulation, and compatibility with different fabrication approaches.

## Graphical abstract

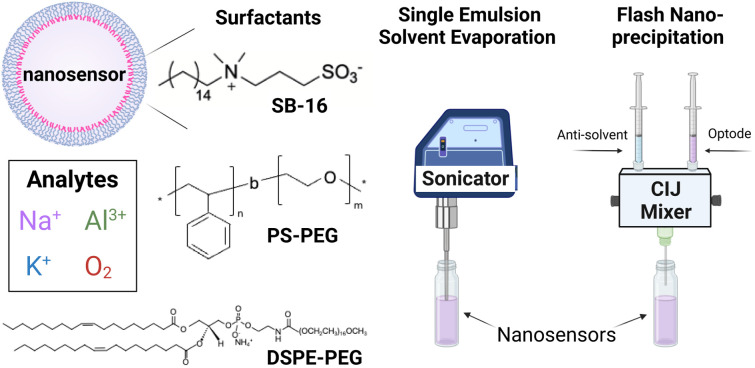

## Introduction

Optical sensors, as implied by their name, use an optical readout mechanism to report changes in analyte concentration. Examples include techniques such as Surface Enhanced Raman Spectroscopy, Localized Surface Plasmon Resonance, colorimetry, absorbance, and luminescence.^1^ These readout methods can be integrated with sensing components to develop sensors with diverse functionalities. One notable class of these sensors is bulk optode membranes, which can combine the capabilities of traditional ion-selective electrodes with readouts from optical reporters.^2^ These sensors are adept at detecting changes in analytes such as oxygen,^3,4^ ions,^5–7^ and pH.^8,9^ Another promising sensing format is nanosensors, which are exciting because they have small sizes (∼100 nm) and a high surface area to volume ratio which means that they can be deployed into biological systems while being minimally invasive and have faster readout times than bulk optode films. Like its optode counterpart, by employing custom or commercial sensing components into the nanosensors, they can be tuned to measure a plethora of analytes such as pH,^10^ iron,^11^ potassium,^12–14^ and calcium.^15–18^ This makes nanosensors a valuable analytical tool to study biological phenomena like electrolyte regulation in animals like mice,^19^ or metabolic activity of microbes such as bacteria or fungi.^20^

As the field of sensing continually evolves, advancements in ionophores and reporters (such as chromoionophores, solvatochromic dyes, *etc*.) have contributed towards the development of more robust nanosensors that are not just more selective to their analyte of interest, but ones that can also overcome bio-imaging challenges like depth penetration or autofluorescence.

While much has been done to improve sensor performance, many in the field are evaluating the influence of surfactants on nanosensor stability, particle sizes, and selectivity. As far back as 1996 Torre *et al.* highlighted that poly(ethylene oxide)-based nonionic surfactants such as Brij-35 and Triton X-100 can influence the response of membrane-type ion selective electrodes (ISEs).^21^ Furthermore, Malinowska *et al.* showed that similar nonionic surfactants decreased the selectivity of sodium, potassium, and calcium-selective membranes.^22^ According to their theoretical model, they attributed this decrease to the concentration of the surfactant during calibration and the partition coefficient of the surfactants into the ion-selective membranes. In the realm of nanosensors, Corrie and coworkers, recently developed a ratiometric organosilica oxygen nanosensor to spatially and temporally profile oxygen profiles in bulk and static bacterial cultures. They used a polyethylene glycol (PEG) chain to enhance the biocompatibility and colloidal stability of their sensors.^23^ Similarly, Wang *et al.* used Pluronic F-127 to develop a silica type dual nanosensor capable of monitoring pH and O_2_ in cell's cytosol. They showed that Pluronic was able fabricate nanosensors and conjugate other fluorophores to the sensor surface, FITC for pH detection in this instance.^24^ Xie and Bakker also highlighted the utility of Pluronic F-127 in the fabrication of sodium-selective nanospheres. They hypothesized that unlike PEG which merely acted as a surfactant, Pluronic's poly(propylene oxide) chains were able to embed themselves into the hydrophobic core and help stabilize the particle core leading to smaller particle sizes with lower polydispersity while still providing a sodium response.^25^ Clark's group demonstrated how a surfactant (polyethylene-glycol-lipid or PEG-lipid) could be used to fabricate a biocompatible nanosensor whose physical attributes can be modulated (size and zeta potential) by varying surfactant concentration.^26^ In their paper, Xie *et al.* showed how the introduction of Pluronic F-127 resulted in the formation of sensors smaller than bulk PVC membranes. They use these sensors to evaluate the stability constants of ion-carrier complexes with solvatochromic dyes.^27,28^ Michalska's group also showed that various surfactant types (anionic, cationic and amphiphilic) were not only able to influence the sensitivity of their sensors but also the kinetics of the complex formation.^29^ More recently, Robinson *et al.* further highlighted the impact of surfactant choice on nanosensor performance. In these reports, it was shown that nonionic, polyethylene-glycol (PEG) based surfactants (Pluronic F-127, Triton X-100, and Brij-35) can partition competing ions into the sensing phase thus reducing the sensors’ selectivity to the analyte of interest.^30^ These findings are impactful as traditional ionophore-based optical sensor theory, originally developed for optodes, does not consider the role of surfactants and their impact on nanosensor performance. Additionally, they showed that a zwitterionic surfactant, sulfobetaine 16 (SB-16), can improve the selectivity of cationic sensors to the analyte of interest.^30^ To demonstrate this, they used a thin-film voltammetry approach to ion sensing in tandem with optical approaches and demonstrated that compared to other surfactants like F-127, Brij35, and Triton X-100, SB-16 improved nanosensor selectivity against competing ions. Using an absorbance-based read-out method, they showed that surfactants such as SB-16 can be used as stabilizers for nanoemulsions. However, they also highlighted that additional work needs to be done to evaluate the feasibility of zwitterionic surfactants in the field of ion sensing.

The adaptation of zwitterionic surfactants into sensors or nanoparticles has been explored by others in various forms. Mao *et al.*, used CHAPS, another zwitterionic surfactant, to fabricate nanoemulsions to evaluate the mass transfer of sensing components into ion-selective membranes.^31^ Wang *et al.* showed that zwitterionic amino acids can be used to provide anti-fouling capabilities for silica nanoparticles.^32^ Additionally, Li *et al.* developed a biocompatible fluorescent nanogel capable of bioimaging and drug delivery by copolymerizing a zwitterionic monomer to a carbon dot.^33^ However, all these works predominantly focused on the surfactant's prowess in its antifouling properties, targeted delivery, or mono-valent cation sensing.

In this work, we further explored the utility of SB-16 on nanosensor performance – impacts on different fabrication approaches, analytes of interest, sensing mechanism, and the ability to incorporate nanomaterials into the nanosensor. We compared these metrics against another zwitterionic phospholipid-polymer conjugate (DSPE-PEG) as a reference (see Fig. S1† for chemical structure). The two fabrication approaches we used were single emulsion solvent evaporation (SESE) and flash nanoprecipitation (FNP). Using these techniques, four different sensors were fabricated for measuring sodium, potassium, aluminium, and oxygen. The aluminium (Al^3+^) sensors, fabricated *via* the SESE method, use a liquid–liquid extraction mechanism to detect analyte. The oxygen (O_2_) sensors, fabricated *via* SESE and FNP, operate *via* a collision quenching mechanism which causes their luminescence to change in response to varying oxygen concentrations. Finally, the sodium (Na^+^) and potassium (K^+^) sensors, fabricated using SESE, respond to the analyte by an ion-exchange mechanism. The potassium sensor in this work also had a quantum dot (QD 520) incorporated into the sensing phase to act as a reference signal. The ion sensors were tested for their analyte sensitivity, functional lifetime, and selectivity against other monovalent and divalent cations.

## Experimental

### Materials

Polyvinyl chloride (PVC), bis(2-ethylhexyl) sebacate (BEHS), tetrahydrofuran (THF), dichloromethane (DCM), potassium ionophore 3, Selectophore (KI III), sodium tetrakis[3,5-bis(trifluoromethyl)phenyl]borate, (NaBARF), Chromoionophore III, Selectophore (CH III), 8-hydroxyquinoline (8HQ), Alpha Tocopheryl Acetate or vitamin E acetate (VEA), CdSe/ZnS core–shell type quantum dots, stabilized with octadecyl amine ligands *λ*_em_ 520 nm (QD 520), sodium ionophore X, Selectophore (NaI X), 4-(2-hydroxyethyl)piperazine-1-ethanesulfonic acid (HEPES), sodium acetate, glacial acetic acid, aluminium trichloride hydrate (AlCl_3_), α-d-glucose (G), glucose oxidase from *Aspergillus* niger (GOx), 3-(*N*,*N*-dimethylpalmitylammonio)propanesulfonate (sulfobetaine or SB-16) and 18-gauge needles were all purchased from Sigma-Aldrich (St Louis, MO, USA). Platinum(ii) octaethyl phorphine (PtOEP) was purchased from Frontier Scientific (Logan, UT, USA). 2 M solution (TRIS, 2 M) and 96-well black-walled optical bottom plates were purchased from Fisher Scientific (Waltham, MA, USA). 1,1′-Dioctadecyl-3,3,3′,3′-Tetramethylindocarbocyanine Perchlorate (DiI) was obtained from ThermoFisher Scientific (Waltham, MA, USA). 1,2-Dipalmitoyl-*sn-glycero*-3-phosphoethanolamine-*N*-[methoxy(polyethylene glycol)-750] ammonium salt (PEG-750 or DSPE-PEG) was purchased from Avanti Polar Lipids (Alabaster, AL, USA). Chromoionophore V (CH V) was acquired from Santa Cruz Biotechnology (Dallas, TX, USA). The poly(styrene)-*b*-poly(ethylene oxide), (PS-PEG or PS_1.6k_-*b*-PEG_5k_) was obtained from Polymer Source, Inc. (Montreal, QC, CA). 0.8 μm polyether sulfone (PES) membrane filters were purchased from Pall Corporation (New York, NY, USA). Ultrahigh purity nitrogen gas and compressed air were purchased from Matheson (Denver, CO, USA). 10 mm pathlength quartz cuvette with rubber septa seal cap was purchased from Starna Cells (Atascadero, CA, USA).

### Optode and nanosensor fabrication

#### Sodium and potassium sensor optodes

Both optodes and sensors were fabricated by the procedure outlined by Sodia *et al.*^11^ In short, the PVC and BEHS were mixed in a 2 mL glass vial and vortexed till homogeneous. In a separate 2 mL glass vial each sensor's sensing components were added – the sodium sensors’ optode comprised of CH III (in THF), NaBARF and NaI X, while the potassium sensor had CH V (in THF), NaBARF, QD-520 (in THF) and KI III (exact masses of each component can be found in Table S1 of the ESI†). The sensing components were raised to 250 μL THF before being added to the PVC-BEHS mixture. Afterward, 250 μL DCM was added, and the mixture was vortexed for 1 min before storage at 4 °C.

#### Oxygen sensor optodes

Both SESE and FNP optodes used PtOEP and DiI. The optode used in the SESE approach was modelled after the procedure outlined by Saccomano *et al*.^34^ The two exceptions are that instead of PtOEPK and DiD, PtOEP and DiI were used respectively. The optode used in the FNP approach was similar to that described by Mendonsa *et al.*,^35^ the difference being the dyes used and the final volume of the optode. The exact values of each sensing component can be found in Table S2 of the ESI.†

#### Aluminium sensor optode

The Al^3+^ optode and sensors were prepared as described by Sodia *et al*.^11^ The exact values of the sensing components, buffer, and surfactants can be found in Table S1 of the ESI.†

#### Nanosensor fabrication

For the SESE method, the nanosensors were fabricated as outlined by Saccomano *et al.*^34^ To summarize the procedure, the surfactant of choice was added to an 8 mL shell vial, raised in the 5 mL of buffer (pH 7.4), and sonicated for 30 s at 20% intensity with a probe tip sonicator (Branson Ultrasonics, Brookfield, CT, USA). The mixture was sonicated a final time for 3 min at 20% intensity while simultaneously injecting 100 μL of optode. Finally, the sensors were then filtered through a 0.8 μm PES membrane filter and stored in a cool, dark place till further use.

For the sensors fabricated *via* FNP, we followed the procedure outlined by Mendonsa *et al.*^35^ with two exceptions to this procedure. The first is that for the SB-16 sensors, the surfactant was added to the PBS (anti-solvent stream), and the optode in the solvent stream. The second difference is that the sensors were air-dried under a gentle air stream for 25 minutes after being left to stir for 10 min post-fabrication. These sensors were also stored in a cool, dark place till further use. See Table S2 in the ESI† for exact volumes and masses for each component.

### Nanosensor characterization

#### Ion sensors

A Synergy H1 microplate reader from Biotek (Winooski, VT, USA) was used to profile the selectivity and sensitivity of the ion sensors. The sodium and potassium sensors were independently tested against Na^+^, K^+^, Ca^2+^, and Mg^2+^ dilution sets with concentrations ranging from 2 μM to 2 M (made in HEPES/Tris (H/T) buffer at pH 7.4). The nanosensors and the analytes were mixed in a 1 : 1 volume ratio in a 96-well black-walled non-treated clear bottom optical plate (Nalgene Nunc International, Roskilde, Denmark) and the fluorescence readout was measured for each nanosensor (585 nm and 650 nm for the sodium sensor, and 510 nm and 710 nm for the potassium sensor). Normally calibration curves are normalized to acid and base endpoints to fully protonate or deprotonate the chromoionophore, but to maintain the zwitterionic nature of the surfactant we decided not to test the sensors at extreme pHs as it would develop a positive or negative charge. As a result, the data was normalized to its respective analytes’ response at 1 × 10^−6^ M to ensure an even comparison. GraphPad Prism 10.0.2 software (San Diego, CA, USA) was used fit the data to a 4-parameter logistic equation and to perform statistical analysis. However, for some analytes the sensors overall response was less than 50%, as a result those selectivities and midpoint responses were calculated by the extrapolated midpoint generated by the Graphpad Prism. The particle size distribution and *ζ*-potential measurements were obtained on a Brookhaven ZetaPALS (Brookhaven Instruments Corporation, Holtsville, NY) by diluting the sensors to 10% by volume in HEPES/Tris buffer to a total volume of 2 mL. Additionally, to evaluate the QD incorporation into the potassium SB-16 sensors, the sensors were diluted to 20% of their original concentration and loaded onto a lacey carbon grid. The images were acquired on a Tecnai T-12 Transmission Electron Microscope.

#### Aluminium sensors

The aluminium sensors’ sensitivity was profiled similarly to that of the ion sensors as described above. The only exceptions were that the dilutions were made in acetate buffer (adjusted to pH 4.6) and the concentrations ranged from 1 μM to 100 μM. The fluorescence readout at 505 nm was used to generate a calibration curve which was plotted using GraphPad Prism 10.0.2 software.

#### Oxygen sensors

To profile the response of the oxygen sensors a gas bubbling system was used to vary the concentration of oxygen from 0% to 21% dissolved oxygen concentration at 5675 ft (elevation of Golden, CO, USA). The sensors were excited with a 405 nm LED source and the emissions were observed *via* an Avantes StarLine spectrometer with a 200 μm slit width purchased from Avantes (Lafayette, CO, USA). The data points were averaged at 580 ± 2 nm and 650 ± 2 nm for DiI and PtOEP respectively. To build a Stern–Volmer calibration curve the PtOEP intensity at 0 mg L^−1^ O_2_ (*I*_O_) was divided by the intensity at a given oxygen concentration (*I*). This ratio (*I*_O_/*I*) was then plotted as a function of its respective dissolved oxygen concentration. For the pseudo-Stern–Volmer (pS.V) a similar approach was taken, but the ratiometric intensity (*R*) was used. This ratiometric value was obtained by dividing the PtOEP signal by the DiI signal.

## Results and discussion

Non-ionic polymeric surfactants, such as those based on polyethylene glycol *e.g.*: Pluronic F-127 or PEG, have previously been used to fabricate most ionophore-based optical sensors, as they excel in forming and stabilizing nanoparticles. But as highlighted by Robinson and others,^21,22,30^ the aforementioned surfactants can impact the sensor response as they can aid in the partitioning of competing analytes into the sensing phase thus impacting metrics such as sensitivity and selectivity.^30^ As a result, we decided to explore zwitterionic surfactants as a promising alternative to the aforementioned PEG-based surfactants and explore how these surfactants impact other mechanisms and fabrication approaches. In this work, we used two zwitterionic surfactants, a phospholipid-polymer conjugate (DSPE-PEG) and a sulfobetaine derivative (SB-16), to fabricate sensors with three different sensing mechanisms – an oxygen sensor that responds *via* collisional quenching, an aluminium sensor which uses a liquid–liquid extraction mechanism and finally sodium and potassium sensors which use an ion-exchange mechanism.

**Fig. 1:**
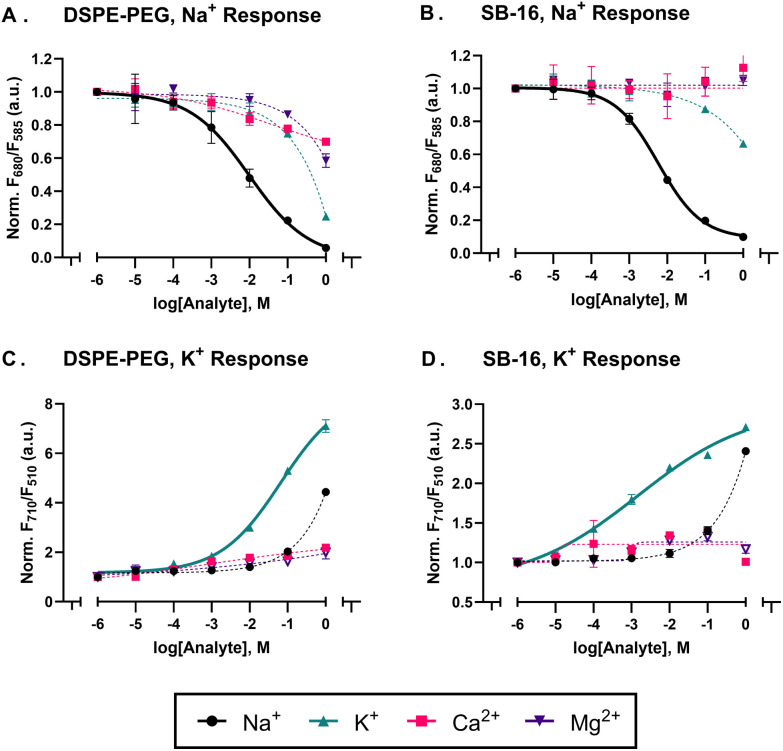
(A) and (B) shows the response of sodium sensors against competing ions (K^+^, Ca^2+^, Mg^2+^) fabricated with DSPE-PEG and SB-16 respectively. The data was plotted as a ratio of the CH 3 emissions at 650 nm divided by the emissions 585 nm. (C) and (D) shows the response of the potassium sensors against competing ions (Na^+^, Ca^2+^, Mg^2+^) fabricated with DSPE-PEG and SB-16 respectively. The data was plotted as a ratio of the CH V and QD 520 emissions at 710 nm and 510 nm respectively. Note that all the data was normalized against the analyte response at 1 × 10^−6^ M. Where not visible the error bars are smaller than the data points (*n* = 3).

Using the SESE method, we fabricated sodium and potassium selective sensors with DSPE-PEG and SB-16 and tested them for their sensitivity and selectivity. As outlined in the methods section, both sodium and potassium sensors were added to Na^+^, K^+^, Ca^2+^, and Mg^2+^ analyte with each analyte's concentration ranging from 1 μM to 1 M – a concentration range that encompasses and exceeds the concentrations of electrolytes in most biological systems.^36–38^ The DSPE-PEG and SB-16 sodium sensors had similar sensitivities, with mid-point responses (log EC50) values of −2.01 and −2.24, respectively. However, their selectivity coefficients differed greatly. As expected, the PEG-based sodium sensors showed a greater affinity towards K^+^ analyte than its SB-16 counterpart, as seen in Fig. 1 with selectivity coefficients (log *K*^Osel^_Na,K_) with values of −0.59 and −8.36 for DSPE-PEG and SB-16, respectively (see Table S3 in the ESI† for other *K*^Osel^ values). On the other hand, the DSPE-PEG and SB-16 potassium sensors had a log EC50 value of −1.18 and −2.83, respectively towards K^+^ ions and a log *K*^Osel^_K,Na_ of −6.25 and −6.90 respectively (see Table S3 of the ESI† for other *K*^Osel^ values). At a glance, the SB-16 potassium sensors appear to have a linear response to analyte, which could allude to a rate-limiting step in the ion-exchange process as highlighted by Kisiel *et al*.^39^ To evaluate this, we expanded the analyte range from a low point of 1 μM to 1 nM to see if the sensors still exhibit a linear response (which would imply they operate exhaustively) and conducted a kinetic study to see if the response changed over time. As seen in Fig. S2,† we obtained a sigmodal response that did not change over the course of 18 hours.

As expected, both, sodium and potassium sensors made with SB-16 showed better selectivity against competing ions over its DSPE-PEG counterpart (see Fig. 1). It is worth noting, that due to the sensors’ minimal response to off-target analytes, the four-parameter hill curve used to fit the data is poorly constrained and results in selectivity coefficients greater than −3.0 (see Nanosensor characterization). However, it's clear from the data in Fig. 1, that the sensors are selective to their target analyte over their off-target counterparts. The poorer selectivity of the DSPE-PEG sodium sensors can be attributed to the surfactant enhancing the extraction of competing ionic analytes into the sensing phase. As highlighted by Malinowska *et al.*,^22^ poly(ethylene oxide) based surfactants can wrap around alkaline metals within the sensing phase due to their polyether chains and as a result impact selectivity.^22,40^ However, given our sensing format differs from that of Malinowska *et al.*^40^ (nanosensor *vs.* ISE) we ran a control with no ionophore to better understand the role of the surfactant. As seen in Fig. S3† the DSPE-PEG sensors showed a response to increasing ion concentration but did not selectively partition any specific analyte into the sensing phase, while its SB-16 counterpart did not respond to the increase in ion concentration. This implies that in our sensing format, the PEG-based surfactant acts as an ion-exchanger rather than an ionophore. It is also worth noting, that while the surfactant can influence selectivity to a certain degree,^27^ a bigger role is played by the ion-ionophore interaction as determined by the ionophores’ binding constant. This is best evidenced in the case of the potassium sensors in Fig. 1, where the surfactant does not impact the selectivity coefficient nearly as much as its sodium counterpart.

In terms of physical properties, we characterized both DSPE-PEG and SB-16 sensors’ functional lifetime and colloidal stability. As seen in Fig. S4,† there is no change in the normalized ratiometric response of the SB-16 sodium sensor to Na^+^ over the course of 5 days, thus implying good functional lifetime, while the similar particle size distributions and *ζ*-potential values of the SESE sensors fabricated with DSPE-PEG and SB-16 suggest good colloidal stability (for more information see Table S4†).

Given the emergence of nanosensors as a tool to study biological systems, we also evaluated the impact of SB-16 sensors on cell viability *via* a CCK-8 assay. To do this the yeast cells were incubated overnight with wort (a nutrient-rich complex media used to grow brewing yeast cells), sodium nanosensors, and the CCK-8 solution with endpoint and absorbance readings taken across two days as per the procedure previously established by our group.^34^ As seen in Fig. S5,† there was no significant change in absorbance value across two days for the SB-16 and DSPE-PEG sensors showing no negative effects on cell viability. This result was expected as the zwitterionic surfactants/polymers used to make these sensors are known to have low cytotoxicity^41–43^ and are used in drug delivery.^44,45^

Another key attribute we evaluated was SB-16's ability to incorporate other nanomaterials into the sensing phase. The sodium sensor, which uses CH III as the optically active moiety with emission peaks at 585 nm and 680 nm is intrinsically capable of outputting ratiometric measurements. The potassium sensors that use CH V as the transducing moiety have only one emission peak at 710 nm, prompting us to add a quantum dot (QD 520) into the sensing phase for ratiometric measurements to be acquired from 710 nm (CH V) and 510 nm (QD 520). The emission of the QD 520 will be gated by the shift in the absorbance of the CH V with respect to analyte concentrations, as seen in calibration curves and the emission spectrum of Fig. 1 and Fig. S6,† respectively. This approach also helped us evaluate if SB16 sensors can incorporate nanomaterials into their sensing phase. The TEM results (Fig. S7†) show the QD 520 is within the SB-16 sensor and the dye retention data (Fig. S8†) shows no QD 520 emission in the filtrate thus highlighting good encapsulation of the nanomaterial within the nanosensor. Additionally, it is known that surfactants containing sulphur groups such as SDS can precipitate in the presence of potassium.^46,47^ To evaluate if our sensors were impacted, we mixed our potassium sensors with HEPES/Tris buffer (pH 7.4) and 10 mM KCl solution and measured the particle size distribution at 0 hours and 24 hours. We found there to be no significant change in the particle size or polydispersity over the course of 24 hours (see Table S5†). To summarize, for the purposes of designing sodium or potassium sensors, SB-16 provides the same benefits as its DSPE-PEG counterpart while enhancing sensor selectivity, therefore making them useful in situations where the sensor signal might be influenced by competing analyte.

**Fig. 2:**
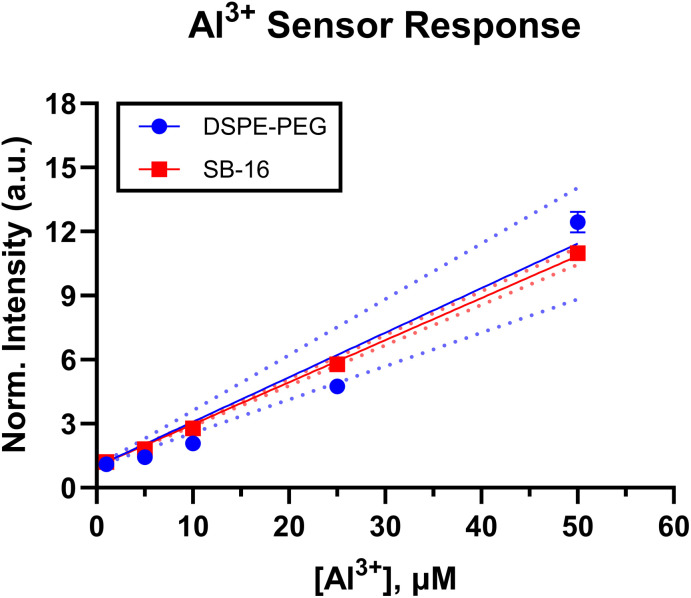
Response of the Al^3+^ sensors made with DSPE-PEG and SB-16 to analyte ranging from 1 μM to 50 μM. While there is a small variation, both responses show a similar trend (two-tailed *t*-test, 95% C.I, *p* = 0.1134 > 0.05). Note, the data was normalized to the response at 1 μM, also where not visible the errors bars are smaller than the data points (*n* = 3).

In addition to sodium and potassium sensors we also fabricated aluminium sensors *via* the SESE method with both DSPE-PEG and SB16. Unlike the sodium and potassium sensors which have different binding and transducing moieties, this sensor detects analyte based on the liquid–liquid extraction approach.^11^ The optically active ligand, 8 HQ, not only binds to the free aluminium analyte in the solution but also transduces this change through a shift in luminescence or absorbance.^48^ The sensor was tested against aluminium chloride dilutions ranging from 1 μM to 0.1 mM (acetate buffer at pH 4.6). The assay was excited at 365 nm and the emissions were observed at 505 nm using a microplate reader. As seen Fig. S9,† both DSPE-PEG and SB-16 sensors show a similar response to the analyte, with the linear response region showing no significant statistical difference observed for both DSPE-PEG and SB-16 (see Fig. 2). Note, that the data was normalized to the response at 1 μM Al^3+^ to enable a more effective comparison between the responses of the two sensors. This helps to account for any differences in sensor response due to the lack of ratiometric readings. A possible explanation for this lack of difference could be due to the operating pH of the solution. Given, that the analyte is buffered at pH 4.6, it could influence the extraction capabilities of both surfactants thus leading to a similar nanosensor response.^49^

While the sodium/potassium and aluminium sensors are different in terms of response mechanism, they all use NaBARF, a charge balancing additive, to aid in optimizing the nanosensor response to its target analyte while maintaining electroneutrality within the sensing phase. The amount of additive in the optode formulations for the ion sensors had to be increased to fabricate a sensor that selectively responded to the analyte of interest for both surfactants. This increase is understandable, as it has previously been shown that PEG-based surfactants^21,22,30^ can impact ISE selectivity against competing analytes, thus making them more sensitive than SB-16 sensors to analyte. By increasing the concentration of NaBARF in the sensing phase we brought parity between the responses of the PEG and SB-16 sensors, as evidenced by the response of the Al^3+^ sensors in Fig. S10.†

Finally, to test the impact of SB-16 as a surfactant on nanosensors with a fundamentally different sensing mechanism, we fabricated oxygen-sensitive sensors using two different fabrication approaches – SESE and FNP. Unlike the aforementioned sensors, these oxygen sensors operate *via* a collisional quenching mechanism.^50,51^ For, both FNP and SESE O_2_ sensors, we used two dyes – PtOEP, an oxygen-sensitive metalloporphyrin, and DiI, an oxygen-insensitive carbocyanine reference dye. PtOEP has a red-shifted emission peak at 650 nm, while DiI has an emission peak at 580 nm as seen in their emission spectra (Fig. S11 and S12† for SESE and FNP respectively). The sensors were tested under different dissolved oxygen concentrations (0.00, 2.31, 5.90, 11.00, and 21.00% O_2_ for the SESE sensors and 0.00, 2.50, 5.00, 10.00, and 21.00% O_2_ for the FNP sensors) and fitted to a pseudo-Stern–Volmer calibration curve as given by eqn (1), where *R*_O_ is the nanosensors’ ratiometric intensity in the absence of oxygen, *R*_i_ is the ratiometric intensity at a given dissolved oxygen concentration respectively, O_2_ is the dissolved oxygen concentration, and p*K*_SV_ is the pseudo Stern–Volmer constant.1
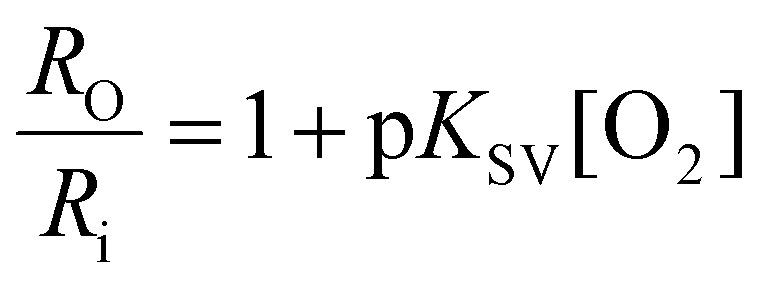


**Fig. 3:**
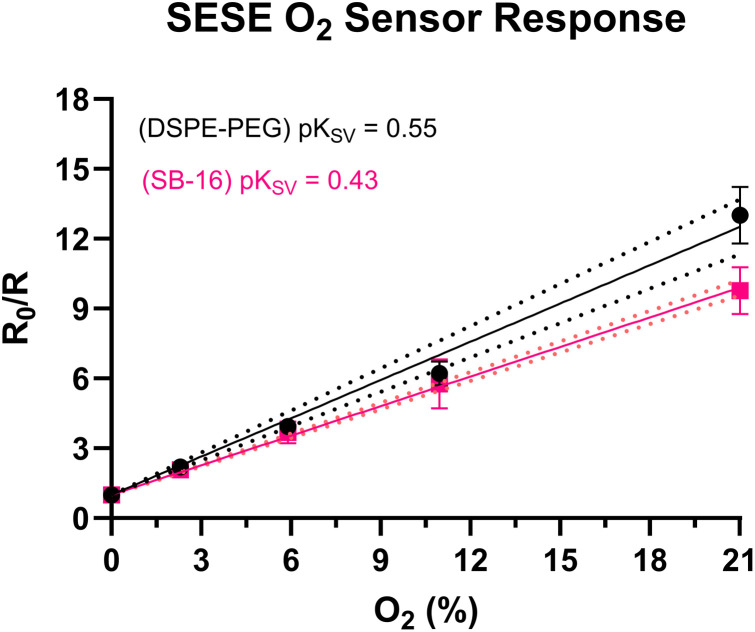
The pseudo-Stern–Volmer calibration curve shows that SESE O_2_ sensors fabricated with DSPE-PEG are more sensitive to oxygen changes than its SB-16 counterpart as evidenced by the higher p*K*_SV_ value (statistical difference verified *via* two-tailed *t*-test, 95% C.I, *p* = 0.0435 < 0.05). Where not visible the error bars are smaller than that of the data points (*n* = 3).

**Fig. 4:**
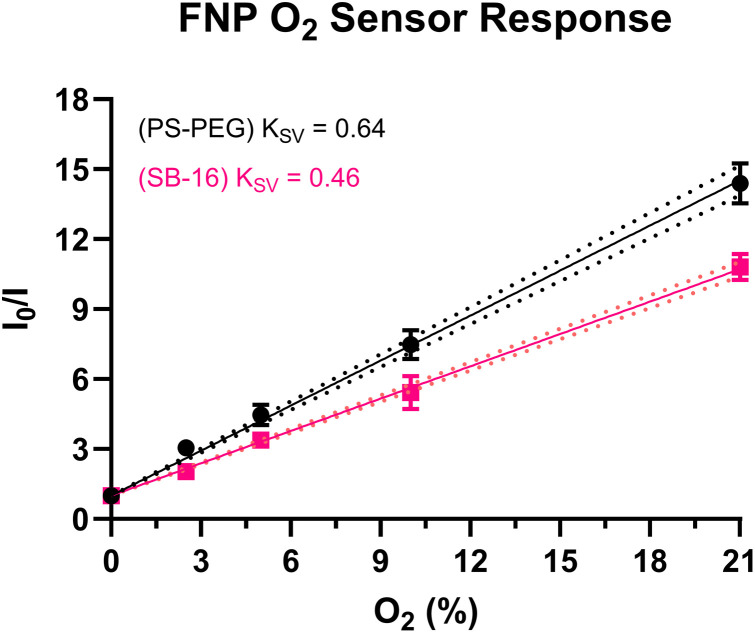
Stern–Volmer response from the O_2_ sensors fabricated using FNP at varying O_2_ concentrations. The sensors made with PS-PEG show a more sensitive response to changes in oxygen than its SB-16 counterpart (statistical difference verified *via* two-tailed *t*-test, 95% C.I., *p* = 0.0037 < 0.05). Both sensors were excited at 405 nm and the emissions observed 650 nm. Where not visible the error bars are smaller than those of the data points (*n* = 3).

As seen in Fig. 3, the SESE-sensor responses seem similar at low O_2_ concentrations (0%–10%), but the p*K*_SV_ shows that the PEG-based sensors (p*K*_SV_ = 0.55) have a higher O_2_ sensitivity than their SB16 counterparts (p*K*_SV_ = 0.43). A two-tailed *t*-test confirms that there is a significant difference between the two responses (95% confidence interval, *p* = 0.0435 < 0.05, see Fig. S13† for Stern–Volmer response). The FNP oxygen sensors’ response (Fig. 4) was profiled *via* the aforementioned gas-bubbling system. These (FNP) sensors also showed a similar trend in that the PEG (PS-PEG) based sensors had a higher sensitivity (*K*_SV_ = 0.64) than its SB-16 counterpart (*K*_SV_ = 0.46), this was confirmed by a two-tailed *t*-test (95% confidence interval, *p* = 0.0037 < 0.05, see Fig. 4, for Stern–Volmer response and Fig. S12† for pseudo-Stern–Volmer response and emission spectra). In terms of physical properties, the SB-16 sensors had a larger average particle size distribution of 402 ± 5.7 nm with a polydispersity of 0.08 ± 0.08 compared to the PEG sensors which had a smaller particle size distribution and polydispersity of 72 ± 0.5 nm and 0.25 ± 0.01 respectively. It should be noted that we did not compare the p*K*_SV_ for the FNP oxygen sensors due to DiI's poor stability. As seen in Fig. S12B,† the emission intensity of DiI increases at 0% O_2_ but is constant between 2.5% to 21% O_2_. This increase in the reference dye resulted in a pseudo-Stern–Volmer graph that had the same oxygen response regardless of the surfactant (see Fig. S12A†). However, this is a problem that can be abated by using dyes with better stability or by just evaluating the response of the oxygen-sensitive dye. The more sensitive response of the PEG-based sensos could be attributed towards the DSPE-PEG impacting the oxygen transfer between the sensor-water interface and therefore leading towards higher sensitivities than the SB-16 O_2_ sensors.^52,53^ Additionally, future work can be done with various zwitterionic and nonionic surfactants to better understand how surfactants can impact oxygen sensitivity specially in the context of nanosensors.

The sensors fabricated in this work showed that compared to DSPE-PEG, SB-16 improved the selectivity of sodium/potassium sensors, while having minimal effect on aluminium and oxygen sensors. This difference could be due to the nature of the sensing mechanisms. In the aluminium and oxygen sensors, the detection and the transduction moieties are the same and less dependent on the ion-exchanger than the sodium/potassium sensors where the charge balancer or ion-exchanger plays a bigger role in mediating the protonation degree of the chromoionophore and thus the sensor response. As highlighted by other groups^27,29,40^ surfactants can influence the response characteristics of sensors. Therefore, it is possible that the response of the sodium/potassium sensors is mediated by surfactants altering the ion-exchange process. While we explored a few ions and sensing schemes, future works could look at a greater array of ions and sensing mechanisms to evaluate if the change in surfactants acts as a detriment instead of a benefit to the sensor.

While the sensors fabricated in this work indicate that SB-16 is a promising alternative to DSPE-PEG in nanosensors for multiple analytes and fabrication approaches, there is room for improvement. The one area of future focus is the pH stability of the SB-16 zwitterionic surfactant to more extreme pH. It is known that zwitterionic surfactants can develop a charge in response to their surroundings’ pH. This effect was seen in the absorbance profile of our SB-16 sodium sensors when tested under acidic conditions (0.1 N HCl) (see Fig. S14†). This change in absorbance was also visibly observable as the wells with SB-16 sensors and acid turned cloudier than the wells with analyte and sensors.

## Conclusions

In this work, we evaluated the utility of SB-16, a zwitterionic surfactant, as an alternative to DSPE-PEG, in the development of nanosensors. To better understand its impact on sensing dynamics, we fabricated sensors with different sensing mechanisms across two different fabrication approaches (SESE and FNP). In general, it was observed that SB-16 was compatible with both fabrication approaches and yielded sensors that had a good functional lifetime (5 days), demonstrated good cell-viability toxicity, and an ability to incorporate other nanomaterials (QDs) into the sensing phase. The Na^+^ and K^+^ sensors that relied on an ion-exchange mechanism, demonstrated better selectivity when fabricated with SB-16 than its DSPE-PEG counterpart. The Al^3+^ (liquid–liquid extraction mechanism) showed no difference in response when tested with DSPE-PEG and SB-16. Meanwhile, the oxygen sensors showed a lowered sensitivity when fabricated with SB-16 than PEG, irrespective of the fabrication approach. While these findings are helpful, future works could look at the development of zwitterionic surfactants that are less pH sensitive. Nonetheless, this work shows that it is worth exploring alternative surfactants to help expand the tools available to develop and improve nanosensors.

## Author contributions

AAM: data curation, formal analysis, investigation, methodology, validation, writing – original draft, writing – review & editing. TZS: data curation, formal analysis, investigation, writing – review & editing. KJC: conceptualization, funding acquisition, project administration, resources, supervision, visualization, writing – review & editing.

## Data availability

The data supporting the findings have been included within this article and the ESI.† Additional information can be provided by the corresponding author (KJC) upon reasonable request.

## Conflicts of interest

There are no conflicts to declare.

## Supplementary Material

AN-149-D4AN00687A-s001
