# Supplementary material for: The impact of zwitterionic surfactants on optode-based nanosensors via different fabrication approaches and sensing mechanisms
Source: Analyst. 2024 Jul 26;149(18):4615–22. doi: 10.1039/d4an00687a (PMC11382340; doi:10.1039/d4an00687a)
Supplement: AN-149-D4AN00687A-s001 [file AN-149-D4AN00687A-s001.pdf]

Supplementary Information (SI) for Analyst.
This journal is © The Royal Society of Chemistry 2024

Supporting Information

The impact of zwitterionic surfactants on optode-based nanosensors via different fabrication approaches and sensing mechanisms

Adrian A. Mendonsa,[a] Tyler Z. Sodia,[b] and Kevin J. Cash*[a,b]

[a] *Chemical and Biological Engineering Department, Colorado School of Mines, Golden, CO, 80401.*
[b] *Quantitative Biosciences and Engineering Department, Colorado School of Mines, Golden, CO, 80401.*
*Corresponding author: kcash@mines.edu

**Table S1**	SESE nanosensor formulation & methods
**Table S2**	FNP Oxygen nanosensor formulation & methods
**Table S3**	Sodium and potassium selectivity coefficients
**Table S4**	DLS and ζ- potential values
**Table S5**	Temporal study on SB-16 potassium sensors and potassium analyte

**Fig. S1**	Surfactant chemical structures
**Fig. S2**	Potassium nanosensor response kinetics over 18 hours
**Fig. S3**	Ionophore-free nanosensor response
**Fig. S4**	Functional lifetime of SB-16 sensors
**Fig. S5**	Measuring cell viability response of DSPE-PEG & SB-16 sensors
**Fig. S6**	Absorption spectra of sodium sensors (PEG and SB-16) under different analyte conditions
**Fig. S7**	TEM images of quantum dots in SESE SB-16 sensors
**Fig. S8**	Rentability of the QD 520 in the SB16 potassium
**Fig. S9**	Response of the aluminum sensors to aluminum analyte
**Fig. S10**	Effect of increasing NaBARF on the response of the aluminum nanosensors
**Fig. S11**	Emission spectra of the (DSPE-PEG and SB-16) SESE oxygen sensors
**Fig. S12**	Calibration curve and emission spectra for oxygen sensors fabricated with FNP
**Fig. S13**	Stern-Volmer response of oxygen sensors fabricated via SESE
**Fig. S14**	Absorption spectra of sodium sensors (PEG and SB-16) under different analyte conditions

Table 1: SESE Nanosensor optode formulation & Methods

Optode components	Sodium Sensors	Potassium Sensors	Aluminum sensors	Oxygen sensors
PVC	15 mg	15 mg	15 mg	15 mg
BEHS	33 µL	33 µL	33 µL	33 µL
NaBARF	1.5 mg	3.5 mg	4mg	-
NaI X	1.0 mg	-	-	-
CH III	0.25 mg	-	-	-
KI III	-	3.0 mg	-	-
CH V	-	0.375 mg	-	-
QD 520 *	-	0.4 mg	-	-
8 HQ	-	-	8 mg	-
PtOEP	-	-	-	0.5 mg
DiI	-	-	-	0.2 mg
THF	250 µL	250 µL	250 µL	250 µL
DCM	250 µL	250 µL	250 µL	375 µL
**Optode per NS batch**	**100 µL**	**100 µL**	**100 µL**	**125 µL**

* The QD 520 was incorporated into the optode as per the procedure by Ruckh et. al [1]. In short, in a 1.5 mL Eppendorf tube, 120 µL ethanol was added to 0.2 mg QD (80 µL of 2.5 mg/mL in THF) and was vortexed for 1 min. After the initial mix, the tube was loaded onto a centrifuge and spun for 5 mins at 8000 RCF. The supernatant was extracted and discard. 80 µL of 3.3 mM dodecanthiol in THF was added to the pellet and then vortexed for 1 min to make a stock of 2.5 mg/mL QD 520 in “thiol-THF”. Two of these stocks were used in one potassium optode.

Table 2: FNP Nanosensor optode formulation & Methods

Optode components	"PEG" Sensors	SB-16 Sensors
PBS (anti-solvent)	0.525 mL	0.525 mL
PS-b-PEO	2.5 mg	-
SB-16 *	-	2.5 mg
VEA	2.3 mg	2.3 mg
PtOEP	0.1 mg	0.1 mg
DiI	0.08 mg	0.08 mg
THF	0.500 mL	0.500 mL
PBS (quench bath)	4.5 mL	4.5 mL

* The SB-16 was dissolved in PBS buffer (pH 7.4) at a concentration of 5.0 mg/mL and then added to the antisolvent stream.

Table 3: Na and K Sensors mid-point response and selectivity coefficients

Log EC50	Na^+	K^+	Ca^{2+}	Mg^{2+}
**Na Sensor (DSPE-PEG)**	-2.008	6.179	-1.423	5.581
**Na Sensor (SB 16)**	-2.239	6.122	> 10	> 10

Log EC50	Na^+	K^+	Ca^{2+}	Mg^{2+}
**K Sensor (DSPE-PEG)**	5.074	-1.179	-3.008	26.92
**K Sensor (SB 16)**	4.057	-2.832	-4.957	-2.99

$\log K_{Na,x}$	Na^+	K^+	Ca^{2+}	Mg^{2+}
**Na Sensor (DSPE-PEG)**	-	-8.187	-0.585	-7.589
**Na Sensor (SB 16)**	-	-8.361	> - 10	> -10

$\log K_{K,x}$	Na^+	K^+	Ca^{2+}	Mg^{2+}
**K Sensor (DSPE-PEG)**	-6.253	-	1.829	-28.099
**K Sensor (SB 16)**	-6.889	-	2.125	0.158

Table 4: DLS and ζ-potential values

DLS Values	"PEG" Sensors		SB-16 Sensors	
	(Size, nm)	Polydispersity	(Size, nm)	Polydispersity
SESE Na Sensors	122 $\pm$ 3.4	0.17 $\pm$ 0.02	205 $\pm$ 1.2	0.28 $\pm$ 0.01
SESE K Sensors	120 $\pm$ 3.6	0.19 $\pm$ 0.02	182 $\pm$ 2.7	0.19 $\pm$ 0.01
FNP O_2 Sensors	72 $\pm$ 0.5	0.25 $\pm$ 0.01	402 $\pm$ 5.7	0.08 $\pm$ 0.08

ζ-potential	"PEG" Sensors		SB-16 Sensors	
	ζ-Potential (mV)	Mobility (μ/s)/(V/cm)	ζ-Potential (mV)	Mobility (μ/s)/(V/cm)
SESE Na Sensors	-23.9 $\pm$ 1.9	-1.87 $\pm$ 0.15	-28.0 $\pm$ 0.6	-2.19 $\pm$ 0.05
SESE K Sensors	-17.5 $\pm$ 2.4	-1.37 $\pm$ 0.19	-49.8 $\pm$ 1.1	-3.89 $\pm$ 0.08
FNP O_2 Sensors	-4.9 $\pm$ 0.8	-0.38 $\pm$ 0.06	-13.6 $\pm$ 1.4	-1.06 $\pm$ 0.11

Table 5: Temporal study on SB-16 potassium sensors stability in potassium analyte

	Sensor in HEPES/Tris		Sensors in 10 mM KCl	
	(Size, nm)	Polydispersity	(Size, nm)	Polydispersity
**Day 1**	182 $\pm$ 1.7	0.17 $\pm$ 0.02	190 $\pm$ 1.6	0.16 $\pm$ 0.03
**Day 2**	178 $\pm$ 3.2	0.20 $\pm$ 0.02	190 $\pm$ 4.9	0.15 $\pm$ 0.04

To evaluate if the sulphur group on the SB-16 precipitates in the presence of potassium, we studied the colloidal stability of the nanosensor under high (10 mM K^+) and low (HEPES/Tris) potassium concentrations. Post-fabrication, the nanosensors were mixed in equal parts with the analyte solutions (1.2 mL sensors: 1.2 mL analyte) and were sampled over 2 days. At the point of evaluation, the samples were extracted and prepared in triplicate for DLS as described in the methods section. As seen by the data, there was a small decrease in the particle size of the nanosensor left in HEPES/Tris (statistically significant difference, two-tailed t-test, $p = .008 < 0.05$) but this could be attributed to the limitations of the instrument. However, the sensors in the 10 mM KCl solution showed no signs of precipitation or changes in particle size over two days indicating good colloidal stability which is in alignment with our findings in Fig. S2 (no statistically significant difference, two-tailed t-test, $p = .88 > 0.05$).

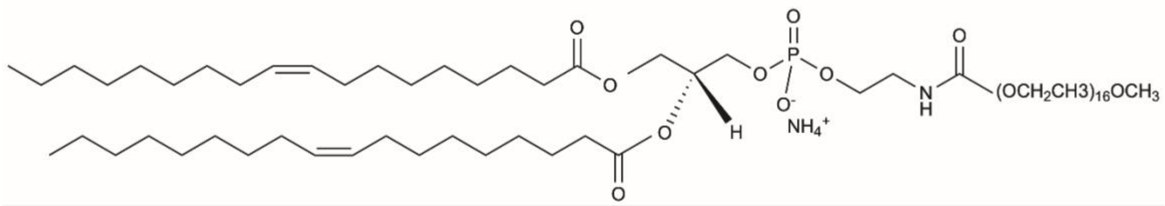

DSPE - PEG

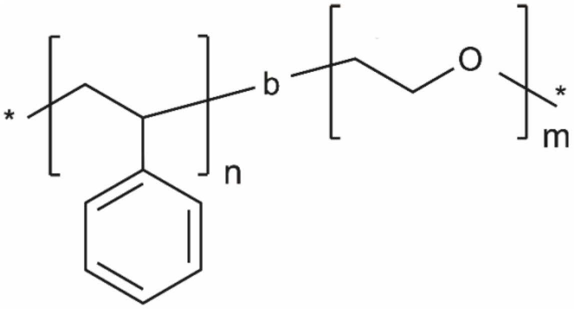

PS-PEG

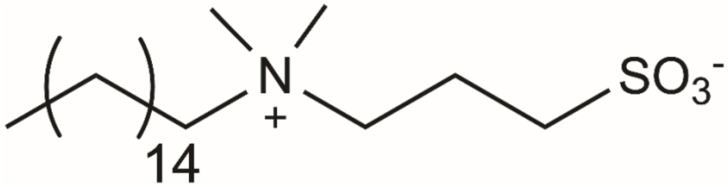

SB-16

Fig. S1: *Chemical structures of the various surfactants used in this work as follows: 1,2-Dipalmitoyl Sn-glycero-3-phosphoethanolamine-N[methoxy(polyethylene glycol)-750] ammonium salt or DSPE-PEG (**top**). Poly(styrene)-*b*-poly(ethylene oxide) or PS-PEG (**middle**). Sulfobetaine-16 or SB-16 (**bottom**)*

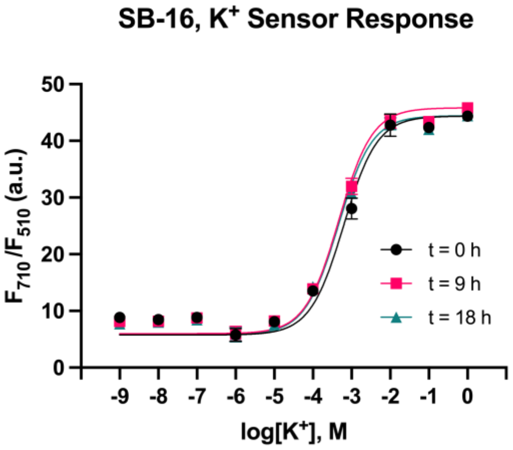

Fig. S2: *Potassium nanosensor were fabricated with SB-16 tested against potassium analyte ranging from 1 nM to 1 M. In the same experiment, a temporal study was done over 18 hours to see if there were any kinetic dependencies in the sensor response. The additional points helped in establishing a sigmoidal calibration curve, while the temporal study showed no change in the sensor response over time. Where not visible the error bars are smaller than that of the data points (n=3)*

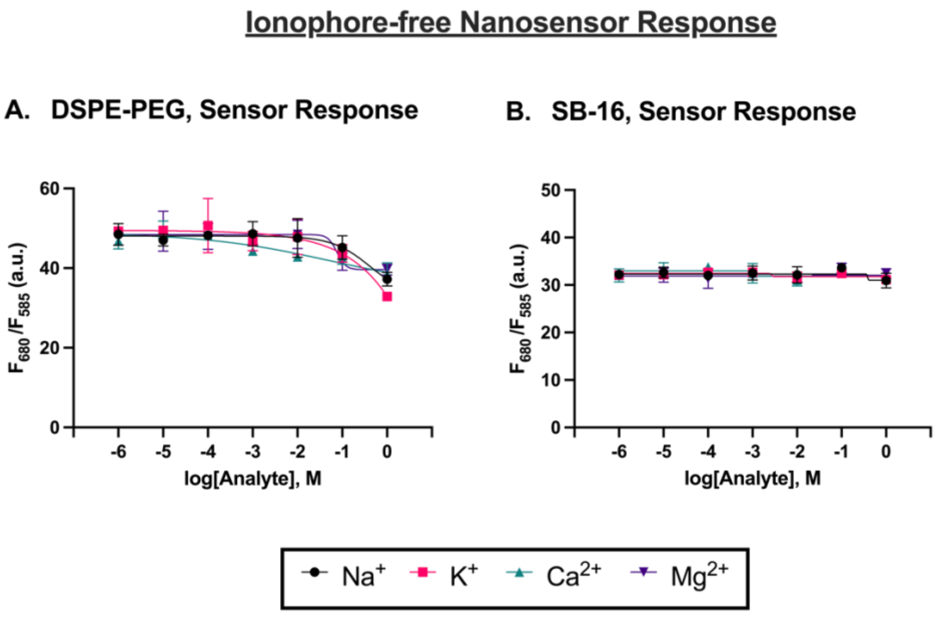

Fig. S3*: **A.** and **B.** Show the response of the sensors were made with all the sodium sensing components sans the ionophore with both DSPE-PEG and SB-16 surfactants respectively. The data showed that DSPE-PEG sensors responded to the increase in the ion concentration for all analytes, unlike it's SB-16 counterpart. This implies that the PEG-based surfactant is playing more of a role as an ion-exchanger than an ionophore in our sensing format. Where not visible the error bars are smaller than that of the data points (n=3)*

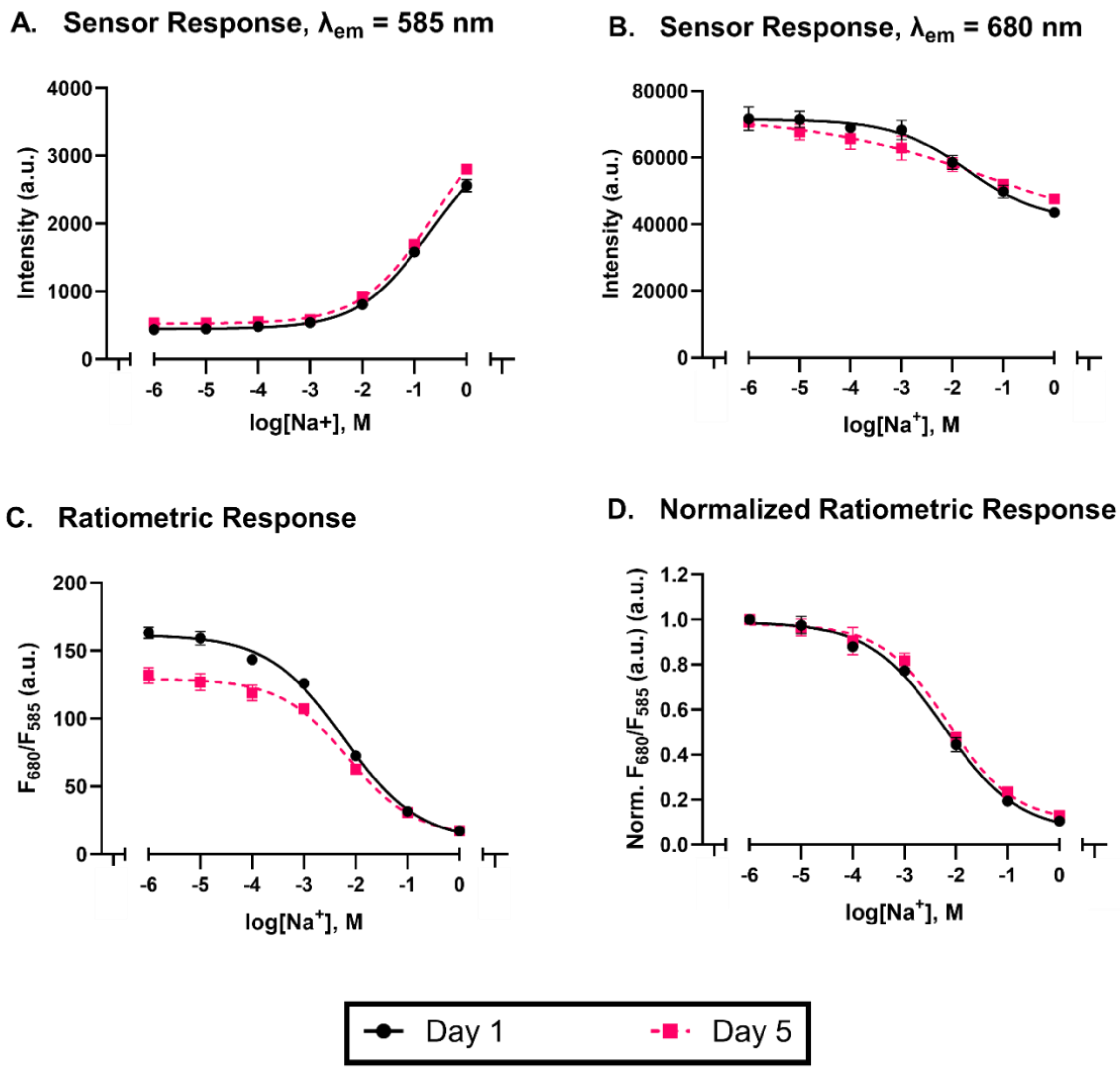

Fig. S4: *Functional lifetime quantified by the response of the sodium nanosensors over the course of 5 days (n=3).* ***A.*** *and* ***B.*** *show the endpoint response of the sodium sensors to analyte at 585 nm and 680 nm respectively.* ***C.*** *The ratiometric response of the sodium sensors seems to show a decrease in signal after 5 days, but this can be attributed to the lowered 680 nm signal on day 5.* ***D.*** *Normalized ratiometric response of the sodium sensors to analyte shows minimal signal degradation over time thus indicating good functional lifetime. This data was normalized against the ratiometric response at* $1x10^{-6}$ *M Na. Note, the ratiometric responses are obtained by dividing the endpoint reading at 680nm by the values at 585 nm. Where not visible, the error bars are smaller than that of the data points.*

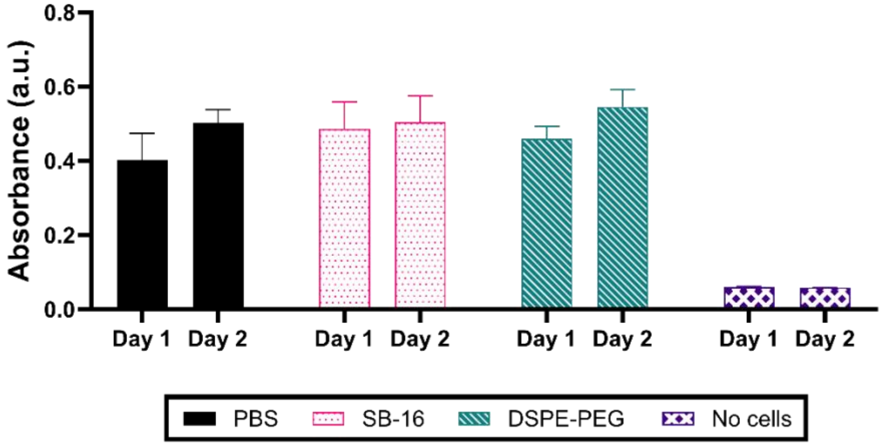

Fig. S5: *CCK-8 Assay monitoring the viability of yeast cells under different conditions, with the absorbance monitored at 460 nm (n =3). The assay was set-up as described by Saccomano et al [2]. As seen in graph above, the cells with SB-16 sodium sensors performed comparable to its PEG and PBS counterparts. If the SB-16 sensors were deleterious to the cells, the lowered metabolic activity would result in the decrease in the production of the orange formazan dye, resulting a lower absorbance at 460 nm.*

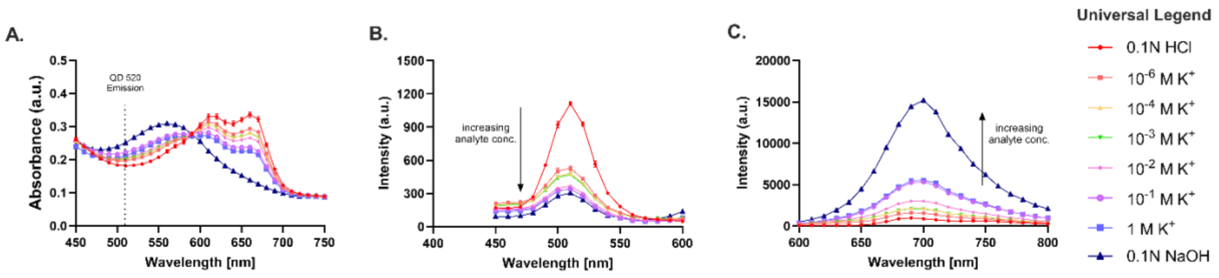

Fig. S6: *Absorbance and emission spectra of the SB-16 potassium sensors, with a quantum dot reference (n=3).* ***A.*** *Absorbance spectra of the potassium sensor. As analyte concentration changes, the absorbance of the CH V in the sensor also changes thus gating the emission of the quantum dot at 510 nm.* ***B.*** *As the concentration of the analyte increases, the absorbance at 510 nm increases, which results in a decrease in the QD emission at 510 nm.* ***C.*** *The CH V emission at 710 nm is opposite to that of the QD 520 response and is linked to the absorption at 550 nm. As result, when the analyte concentration increases, the emission at 710 nm to also increase.*

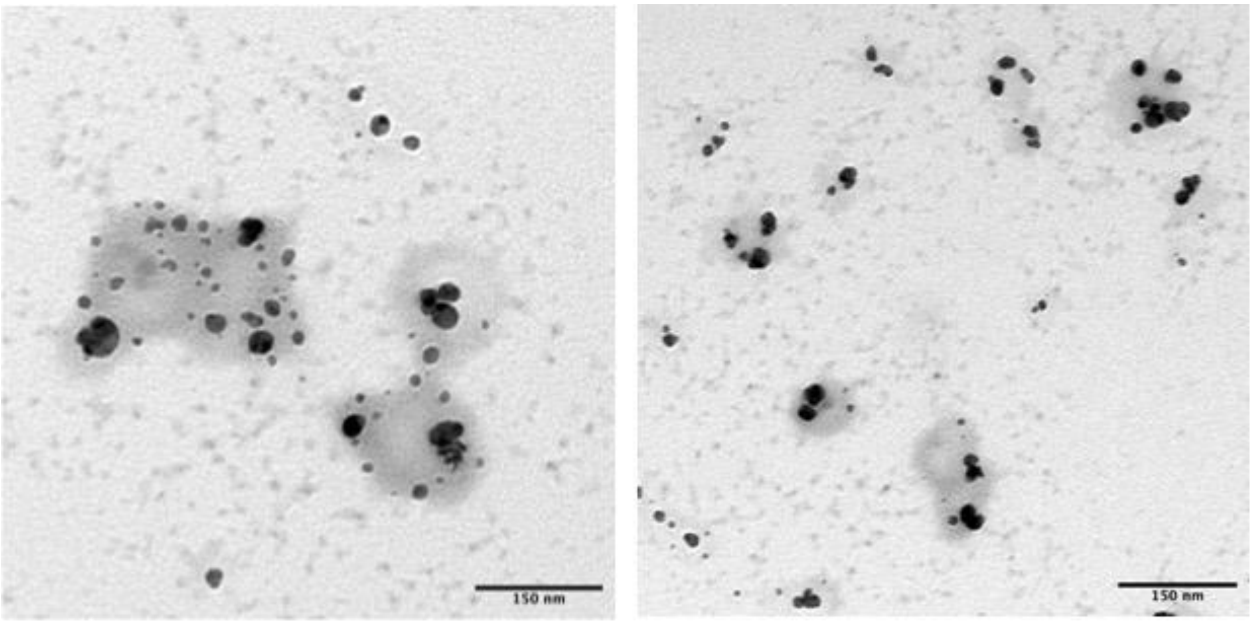

Fig. S7: *TEM images of quantum dots (dark, opaque dots) within potassium nanosensors (larger, translucent grey spheroids). The TEM images show that most nanoparticles are around 150 nm, which is around the ~180 nm reported by the DLS results. Some of the shapes are rather irregular/non-spherical, thus could be due to some of the nanosensors falling apart when loaded onto the TEM lacey carbon grid.*

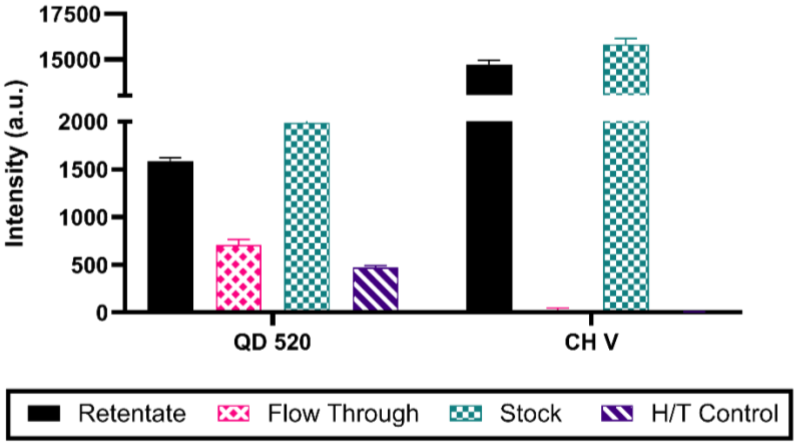

Fig. S8: *Retentability of the QD 520 in the SB16 potassium nanosensors (n=3). To do this, the sensors were concentrated via amicon filters (30 kDa) before the retentate was raised up to the original concentration (equal to that of the stock). The retentate was measured against the flow-through, stock and a HEPES/Tris control. The QD 520 channel retentate showed a 73 % signal retention, while the CH V channel showed a 93% signal retention. The lowered QD 520 signal could be attributed to some of the nanoparticles being caught in the filter.*

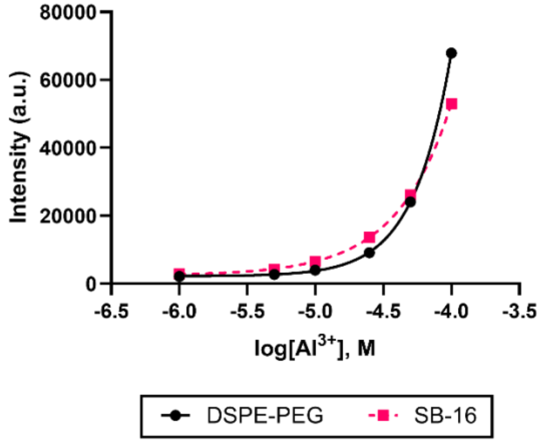

Fig. S9: *Response of the Aluminum sensors to analyte ranging from 1 µM to 0.1 mM (n =3). From this calibration curve, the linear response was generated by evaluating the sensor luminescence for analyte ranging from 1 µM to 50 µM. Where not visible, the error bars are smaller than that of the data points.*

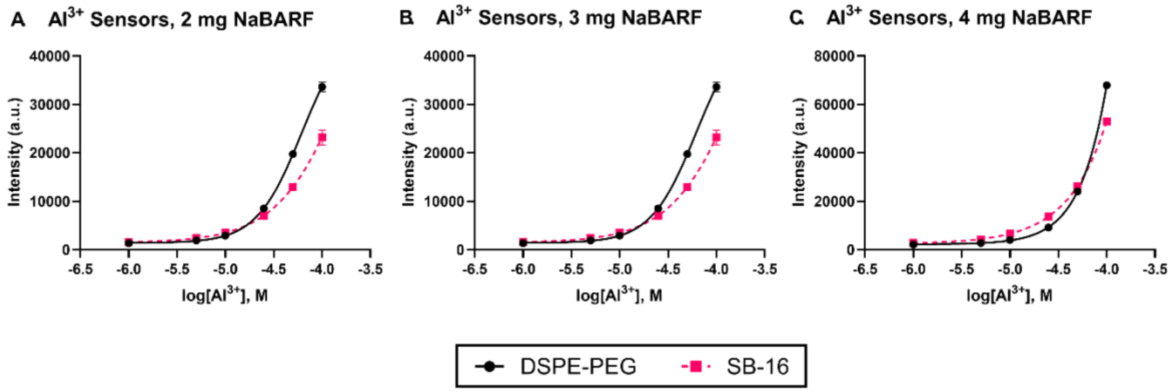

Fig. S10: *Effect of increasing NaBARF on the response of the aluminum nanosensors (n=3).* ***A.*** *shows the luminescence response of the DSPE-PEG and SB-16 sensors with 2 mg of NaBARF in the optode.* ***B.*** *and* ***C.*** *shows the response for 3 mg and 4 mg of NaBARF in the optode.*

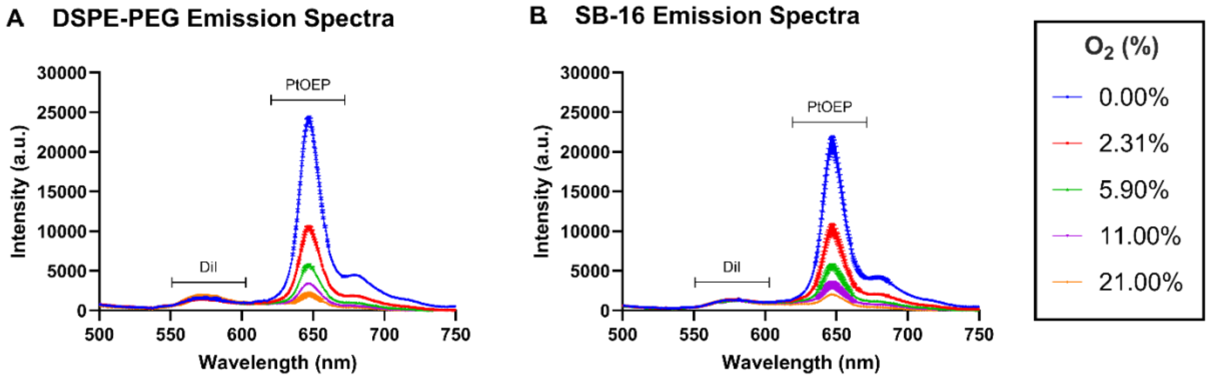

Fig. S11: *Emission spectra of the SESE oxygen sensors in response to different dissolved oxygen concentrations (n=3).* ***A.*** *Emission spectra of the DSPE-PEG based oxygen sensors and* ***B.*** *SB-16 based oxygen sensors. As shown in* ***Figure 3*** *but the pseudo-Stern-Volmers, the DSPE-PEG sensors have a higher sensitivity than its' SB-16 counterpart. Where not visible, error bars are smaller than that of the data points.*

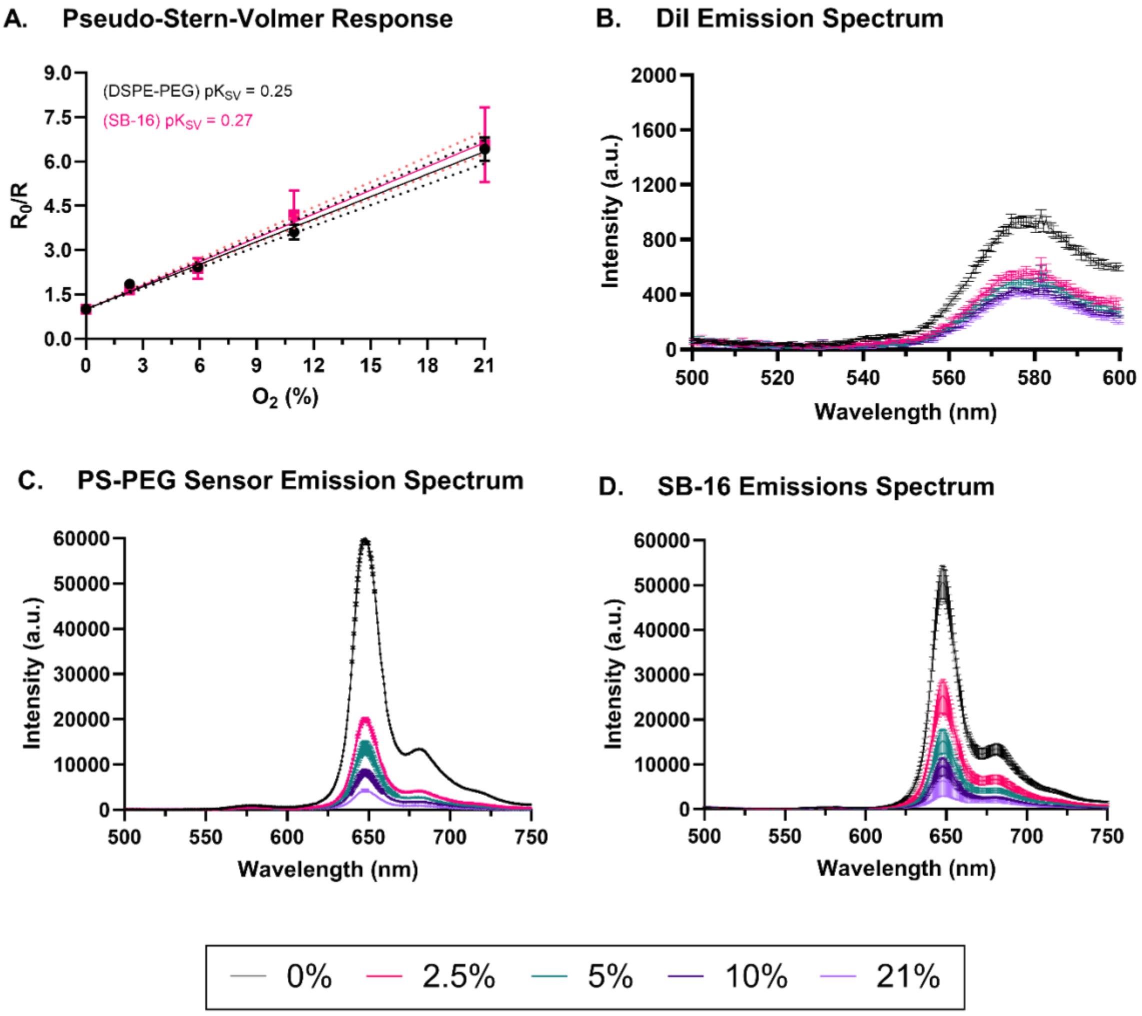

Fig. S12: *Calibration curve and emission spectra for oxygen sensors fabricated with FNP (n=3).* ***A.*** *Shows the pseudo-Stern-Volmer response of both oxygen sensors to varying oxygen concentrations. The* pK_{SV} *show that the responses of both sensors are the same, as validated by a two-tailed t-test (95% C.I,* $p = 0.5691 > 0.05$*). However, this overlap is due to the reference dye not being stable and varying with oxygen concentration.* ***B.*** *Shows the variance in the emission of DiI when exposed to different oxygen concentrations. While the signal is relatively stable between 2.5% and 21% oxygen, at 0 % oxygen there is an increase signal which can impact the slope of the calibration curve as seen in F**ig. S7C.** and **S7D.** shows the emission spectra of the SB-16 and PS-PEG oxygen sensors under various dissolved oxygen concentrations. Where not visible the error bars are smaller than those of the data points (n=3)*

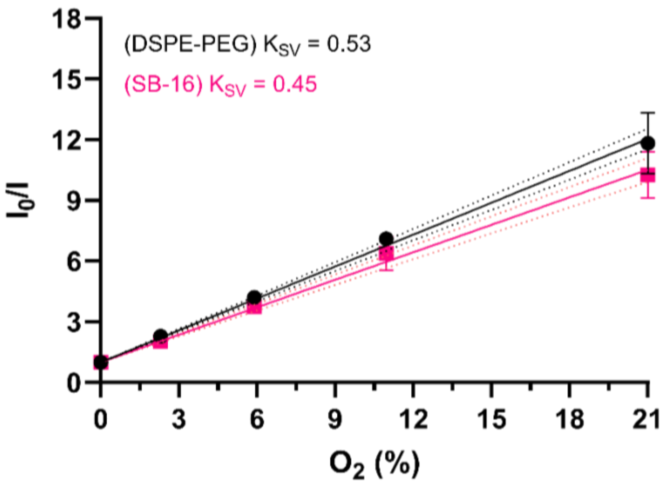

Fig. S13: *Stern-Volmer response of oxygen sensors fabricated via SESE. The sensors were excited at 405 nm and the emissions were observed at 650 nm for each oxygen concentration. The calibration curves show that the PEG sensors are more oxygen sensitive than it's SB-16 counterpart, however, a two-tailed test shows that there is no statistical difference between the responses (95% C.I., p = 0.1676 > 0.05).*

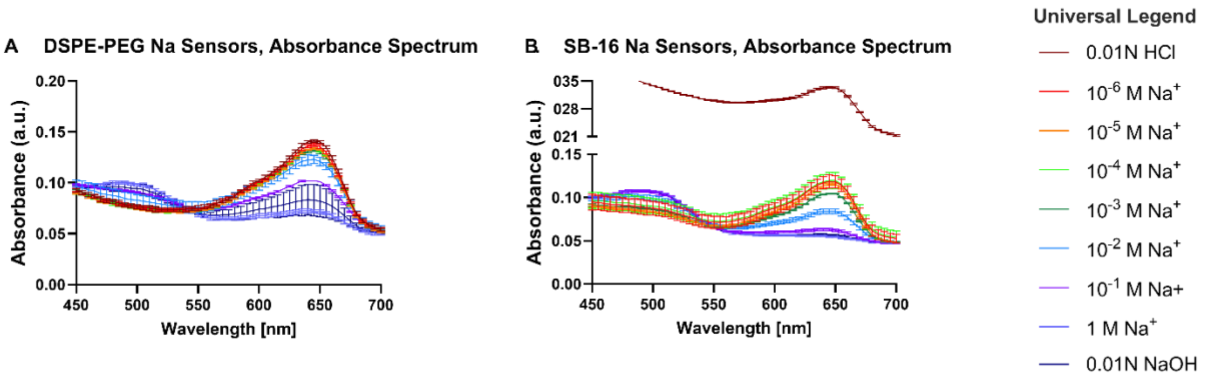

Fig. S14: *Absorption spectra of the sodium sensors under different analyte conditions (n=3).* ***A.*** *Absorption spectra of the DSPE-PEG Na sensors under various sodium dilutions (buffered in HEPES/Tris at pH 7.4) and acid and based points. As expected, the chromoionophore is fully protonated at 650 nm under acid conditions (red line) and deprotonated at 650 nm under basic conditions.* ***B.*** *Absorption spectra of the SB-16 Na sensors under the aforementioned conditions. However, at 650 nm in an acidic environment, the SB-16 sensors seem to have nearly twice the absorbance of its PEG counterpart, thus indicating the sensor might not be completely stable under extreme pH variations.*

References

1 T. T. Ruckh, C. G. Skipwith, W. Chang, A. W. Senko, V. Bulovic, P. O. Anikeeva and H. A. Clark, *ACS Nano*, 2016, **10**, 4020–4030.

2 S. C. Saccomano and K. J. Cash, *Analyst*, 2022, **147**, 120–129.
